# Postoperative pelvic intensity-modulated radiation therapy reduced the incidence of late gastrointestinal complications for uterine cervical cancer patients

**DOI:** 10.1093/jrr/rrz041

**Published:** 2019-06-28

**Authors:** Keisuke Tsuchida, Naoya Murakami, Tomoyasu Kato, Kae Okuma, Hiroyuki Okamoto, Tairo Kashihara, Kana Takahashi, Koji Inaba, Hiroshi Igaki, Yuko Nakayama, Takashi Nakano, Jun Itami

**Affiliations:** 1 Department of Radiation Oncology, National Cancer Center Hospital, 5-1-1 Tsukiji, Chuo-ku, Tokyo, Japan; 2 Department of Radiation Oncology, Gunma University Graduate School of Medicine, 3-39-22 Showa-machi Maebashi, Gunma, Japan; 3 Department of Gynecologic Oncology, National Cancer Center Hospital, 5-1-1 Tsukiji, Chuo-ku, Tokyo, Japan

**Keywords:** uterine cervical cancer, postoperative radiation therapy, intensity-modulated radiation therapy, late adverse effects, ileus

## Abstract

The aim of the study was to compare incidences of late gastrointestinal adverse events and clinical outcomes between 3D conformal radiation therapy (3DCRT) and intensity-modulated radiation therapy (IMRT) after radical hysterectomy for cervical cancer patients. Between March 2007 and May 2014, 73 cervical cancer patients with high-risk prognostic factors (pelvic lymph node metastasis and/or parametrial invasion) underwent postoperative pelvic radiation therapy (RT) after radical hysterectomy. Of these patients, 33 (45%) and 40 (55%) received 3DCRT and IMRT, respectively. Because the gastrointestinal obstruction rate after postoperative pelvic 3DCRT was high, no concurrent chemotherapy was applied until 2015. The median follow-up period for patients with 3DCRT and IMRT was 82 months (6–113) and 50 months (5–74), respectively. There was no significant difference in overall survival (OS) (4-year OS: 85% vs 78%, *P* = 0.744) or disease-free survival (DFS) (4-year DFS: 73% vs 64%, *P* = 0.696) between the two groups. Eleven (33%) and 13 (33%) patients experienced recurrence after 3DCRT and IMRT, respectively. The patients who had vaginal invasion from the postoperative pathological finding more frequently had loco-regional recurrence than the patients who did not have vaginal invasion (2.3% vs 17%, *P* = 0.033). Gastrointestinal obstruction was observed in 9 (27%) and 3 (7.5%) patients for 3DCRT and for IMRT, respectively (*P =* 0.026). Severe gastrointestinal obstruction that required surgery was observed in 6 (19%) patients, all of whom received adjuvant RT by 3DCRT. IMRT could reduce the incidence of late severe gastrointestinal obstruction after postoperative pelvic RT with a non-inferior clinical efficacy compared with 3DCRT.

## INTRODUCTION

Postoperative pelvic radiation therapy (RT) improves outcome in cervical cancer patients with intermediate- or high-risk prognostic factors after radical hysterectomy [1, 2]. However, acute and late toxicities have been noted in the patients treated with radical hysterectomy followed by postoperative pelvic RT. Especially, late gastrointestinal (GI) obstruction is one of the most serious adverse events [1, 3]. Although postoperative pelvic RT is a standard therapy after radical hysterectomy for intermediate- and high-risk early-stage cervical cancer [1, 2], due to the concern for the possible late severe GI toxicities related to the combination of laparotomy and pelvic radiation, and recent emerging positive results from adjuvant chemotherapy alone for intermediate- and high-risk post-hysterectomy cervical cancer [4], several Japanese hospitals have not dared to apply postoperative pelvic RT [5]. Intensity modulated radiation therapy (IMRT) can reduce the volume of the small bowel that receives a high dose of radiation compared with 3D conformal radiation therapy (3DCRT), which theoretically could reduce the incidence of GI obstruction. In the Radiation Therapy Oncology Group (RTOG) Trial 1203, which sought to test the feasibility of delivering IMRT in a multi-institutional study of the treatment of gynecological carcinoma in the post-operative setting, it was revealed that IMRT caused fewer short-term GI adverse events than 3DCRT [6]. However, studies of long-term GI adverse events after IMRT have rarely been reported to date. In this retrospective study, we compared clinical outcomes, incidence of acute and late adverse GI events, and of other adverse events between 3DCRT and IMRT after radical hysterectomy. Additionally, we reported the recurrence patterns of the two radiation methods.

## MATERIALS AND METHODS

This retrospective study was approved by the Institutional Ethical Review Board (Approval No. 2017-091) and was performed in accordance with the ethical standards laid down in the 1964 Declaration of Helsinki and its later amendments.

### Patients

Between March 2007 and May 2014, patients with uterine cervical cancer undergoing radical hysterectomy and shown to have high-risk prognostic factors [pelvic lymph node (LN) metastasis and/or parametrial invasion] underwent postoperative pelvic RT in our institution. Between March 2007 and August 2010, adjuvant RT was performed by 3DCRT with CT planning and thereafter treated by IMRT.

Radical hysterectomy and bilateral pelvic lymphadenectomy was performed for FIGO Stages IB1, IB2, IIA1, IIA2 and IIB cervical cancer patients in our institution. This procedure involves en bloc removal of the uterus, cervix, and parametrial and paracolpium tissues to the pelvic sidewalls bilaterally, with removal of as much of the uterosacral ligaments as possible. The uterine vessels are ligated at their origin, and the proximal third of the vagina and the paracolpium are resected [7].

We excluded patients with positive surgical margins and/or distant metastases, including para-aortic LN metastases from this retrospective analysis.

Concurrent chemotherapy was not administered during the study period because the incidence of ileus was relatively high after postoperative pelvic RT with 3DCRT.

### Radiation therapy

The four box fields of 3DCRT were set up as follows: according to 2D era experience, the following bony landmarks were used for field design using CT image. When patients had tortuous arteries or an extremely large uterus, the field shape was modified to cover an adequate LN drainage area and the entire uterus, based on information obtained from CT. The superior margins were at the intervertebral space between the fourth and fifth lumbar vertebrae, and the inferior margins were at the lower end of the obturator foramen. If the common iliac node was positive for metastasis, the superior margin was placed at the intervertebral space of the second and third lumbar vertebrae. For the AP–PA field borders, the lateral margins were placed at 2 cm lateral to the internal pelvic rim. For the lateral field borders, the posterior border was set in such a way that the entire sacrum was covered. The anterior border of the lateral field was set at a vertical line anterior to the pubic symphysis. The total irradiation dose for 3DCRT was 50 Gy in 25 fractions to the reference point and administered by 15 MV X-rays from Varian linear accelerators (iX, CLINAC, Varian, Palo Alto, California. USA). In the 3DCRT era, no instruction was given to the patients concerning bladder filling.

Regarding the IMRT, the detailed procedure has been described in the reports of our previous studies [8, 9]. A customized immobilization cushion was fabricated to minimize the daily set-up error. Fiducial markers were inserted into the vaginal cuff to visualize it on the CT images [10]. With the patient lying on the immobilization cushion, CT scans with full and empty bladders were taken in order to account for the motion of the vagina as influenced by the contents of the bladder. CT scans of 2-mm slice thickness were taken by an Aquilion LB CT scanner (TOSHIBA Medical Systems, Tokyo, Japan).

The clinical target volume (CTV) was contoured on the individual axial CT slices of each patient. The overall CTV includes both the vaginal cuff/paracolpium CTV and the nodal CTV. The vaginal cuff/paracolpium CTV was contoured in a manner similar to the Radiation Therapy Oncology Group (RTOG) [11] and the Japan Clinical Oncology Group guidelines [9]; cranial margin: 1 cm cranial from the upper part of vaginal cuff metallic marker; anterior margin: posterior border of bladder or retropubic fat pad; posterior margin: anterior part of mesorectal fascia or anterior wall of rectum; lateral margin: medial edge of internal obturator muscle, piriformis muscle, coccygeus muscle and ishial ramus; caudal margin: 3 cm below the upper part of the vaginal cuff metallic marker. The nodal CTV was based on the Japan Clinical Oncology Group Gynecologic Cancer Study Group (JCOG-GCSG) consensus guidelines for the delineation of CTV for pelvic LNs [12]. The nodal CTV included LNs that drain the involved site and adjacent perinodal soft tissue. This included the internal (obturator and hypogastric), external, and common iliac LNs; the presacral LNs and soft tissues also included down to the level of S3. The upper limit of the nodal CTV was the lumbar vertebra (L) 4/5 interspace. If a common iliac LN metastasis was found pathologically, the nodal CTV was extended to the level of the L2/3 interspace. We used the JCOG-GCSG guideline for reference regarding the nodal CTV because it includes adipose connective tissue between the iliopsoas muscles and the lateral surface of the vertebral body, which is not included in the RTOG guideline [11]. This area is also included in an atlas of Taylor *et al.* [13, 14]. The CTV was expanded by 5 mm to create the planning target volume (PTV). For normal structures, the small bowel (contoured as a bowel bag, which is defined as the entire peritoneal space), rectum and bladder (both contoured as a whole organ) and the femoral head were routinely contoured according to the RTOG normal tissue contouring guideline [15]. The planning goals of IMRT were to provide a homogenous PTV dose while minimizing the dose delivered to the small bowel, bladder and rectum. Dose constraints for the PTV and organ at risks are shown in Table 1.

We compared the volumes of the bowel bag receiving greater than or equal to 15 Gy, 30 Gy, 40 Gy or 45 Gy (V_15_, V_30_, V_40_ and V_45_, respectively) and the bowel bag mean dose (Dmean) between 3DCRT and IMRT.

### Evaluation of toxicities

GI, genitourinary (GU), and hematologic (HT) toxicities were assessed according to the Common Terminology Criteria for Adverse Events version 4.0. Late morbidity was defined as morbidity seen >3 months after completion of RT. A GI obstruction of Grade 2 or more was counted as an event. Severe GI obstruction requiring surgery was counted separately.

### Statistical analysis

Differences in clinicopathological factors, dose–volume histogram (DVH) parameters and incidence of acute toxicities between 3DCRT and IMRT were analyzed by the Mann–Whitney U test for quantitative variables and by the Fisher exact test for categorical variables.

The actuarial overall survival rate (OS), disease-free survival rate (DFS), loco-regional control rate (LRC) and incidence of late toxicities were calculated using the Kaplan–Meier method, and differences between groups were compared by the log-rank test. All statistical tests were two-sided, and *P* < 0.05 or a 95% confidence interval (CI) not encompassing 1 was considered as statistically significant.

## RESULTS

Between March 2007 and May 2014, 73 patients were identified who were treated with radical hysterectomy and postoperative pelvic RT. Thirty-three (45%) and 40 (55%) patients received postoperative pelvic RT by 3DCRT and by IMRT, respectively. The median follow-up period for living patients for 3DCRT and for IMRT was 82 months (6–113) and 50 months (5–74), respectively.

Patient characteristics are summarized in Table 2. The characteristics were similar between the two groups; however, relatively more adenocarcinoma patients were included in the IMRT group (but this was not a statistically significant difference). Seven patients had LN metastasis at the common iliac level; therefore, the upper margin of the radiation field for these patients was set as the intervertebral space between the second and third lumbar vertebrae; four and three patients of this seven were in the 3DCRT and in the IMRT groups, respectively.

**Table 1.** Dose constraints of PTV and OAR in IMRT

Structure	Criteria
PTV	Mean dose : 100–105%
	D2%<60 Gy
Bowel bag	D40%<40 Gy
	D1cm^3^<55 Gy
Rectum	D40%<50 Gy
	No volume>55 Gy
Bladder	D50%<45 Gy
	No volume>55 Gy
Femoral head	D20%<30 Gy

IMRT = intensity-modulated radiation therapy, PTV = planning target volume, OAR = organ at risk.

**Fig. 1.**
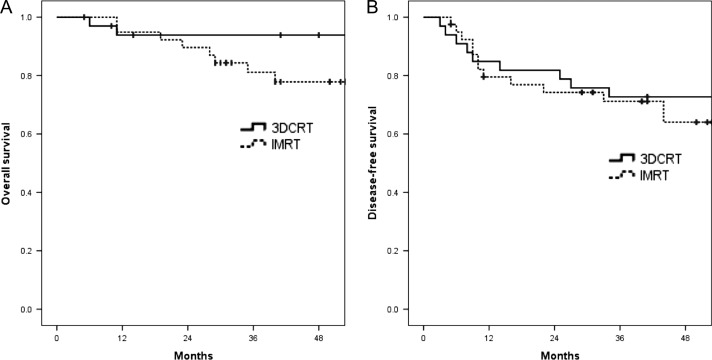
(A) Kaplan–Meier estimates for comparison of overall survival (OS) between 3DCRT and IMRT groups. (B) Kaplan–Meier estimates for comparison of disease-free survival (DFS) between 3DCRT and IMRT groups.

There was no significant difference in OS (4-year OS 85% vs 78%, *P* = 0.744) or DFS (4-year DFS 73% vs 64%, *P* = 0.696) between 3DCRT and IMRT, respectively (Fig. 1A and B).

**Fig. 2.**
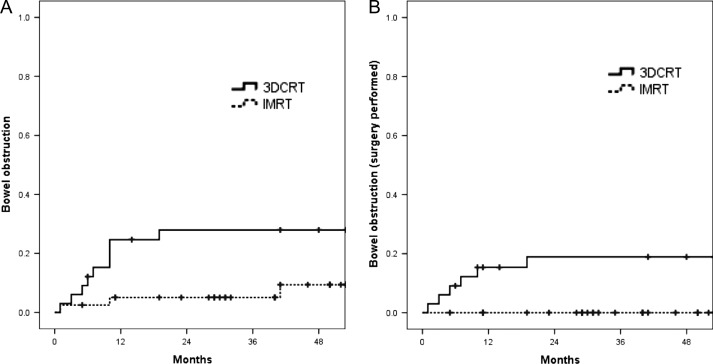
(A) Kaplan–Meier estimates of cumulative incidence curves for any gastrointestinal obstruction between 3DCRT and IMRT groups. (B) Kaplan–Meier estimates of cumulative incidence curves for severe gastrointestinal obstruction that required surgery between 3DCRT and IMRT groups.

The patterns of recurrence are shown in Table 3. Eleven (30%) and 13 (33%) patients had recurrence after 3DCRT and IMRT, respectively. Four (12%) and 2 (5%) loco-regional pelvic recurrences were seen in 3DCRT and in IMRT, respectively, with no statistically significant difference (*P* = 0.270). All loco-regional recurrences were seen in the vaginal cuff or paracolpium. No recurrence was seen in the pelvic LN area. The patients who had vaginal invasion proven in the postoperative pathological findings had more frequently loco-regional recurrence than the patients who did not have vaginal invasion (17% vs 2.3%, *P* = 0.033) (Fig. 2).

**Table 2.** Patients’ clinicopathologic characteristics

	3D-CRT (*n* = 33)		IMRT (*n* = 40)		*P*-value
Age (years)	46 (range 30–66)		42 (range 28–68)		N.S.
Median follow-up period (months)	82 (range 6–113)		50 (range 5–74)		<0.001
	*n*	%	*n*	%	
T stage					N.S.
T1b	8	24	6	15	
T2a	3	9	5	13	
T2b	23	67	29	72	
N stage					N.S.
N0	9	27	9	23	
N1	24	73	31	77	
Inclusion criteria					N.S.
Parametrial invasion + lymph node metastasis	13	39	20	50	
Parametrial invasion	9	27	9	23	
Lymph node metastasis	11	34	11	37	
Histology					N.S.
Sq	22	67	21	52	
AdSq	3	9	4	10	
Ad	8	24	15	38	

3DCRT = three-dimensional conformal radiation therapy, IMRT = intensity-modulated radiation therapy, Sq = squamous cell carcinoma, AdSq = adenosquamous carcinoma, Ad = adenocarcinoma.

**Fig. 3.**
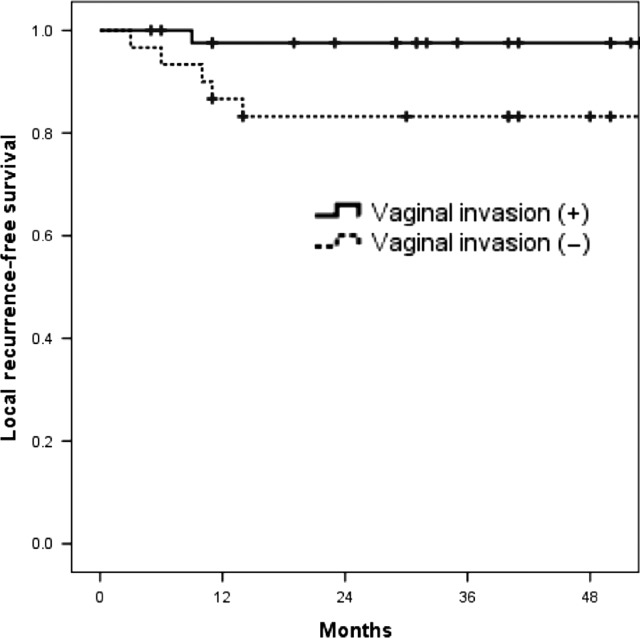
Kaplan–Meier estimates for comparison of local control rate between vaginal invasion–positive patients and vaginal invasion–negative patients.

Among 73 patients, GI obstruction was observed in 9 (27%) and 3 (7.5%) patients for 3DCRT and for IMRT, respectively. Two of 9 were from extended-field patients (28.5%) (1 patient from each of the 3DCRT and IMRT groups). Severe GI obstruction that required surgical intervention was observed in 6 patients, all of whom received adjuvant RT by 3DCRT (18% of 3DCRT patients). Small intestine colon bypass and adhesiolysis surgery were performed for 5 patients and 1 patient, respectively. There was a statistically significant difference in the incidence of all grades of GI obstruction (27% vs 7.5%, *P* = 0.026) and GI obstruction that required surgery (18% vs 0%, *P* = 0.005) between 3DCRT and IMRT, respectively (Fig. 3A and B). (Table 4).

**Table 3.** Patterns of recurrence after 3DCRT or IMRT

	3DCRT (*n* = 33)	IMRT (*n* = 40)	*P*-value
Loco-regional recurrence	4 (12.1%)	2 (5%)	N.S.
Vaginal stump or parametrium	4 (12.1%)	2 (5%)	
Distant recurrence	8 (24.2%)	10 (25%)	N.S.
Para-aortic LN	2 (6.1%)	3 (7.5%)	
Others	6 (18.2%)	7 (17.5%)	
Total	12 (36.4%)	12 (30%)	

3DCRT = three-dimensional conformal radiation therapy, IMRT = intensity-modulated radiation therapy, LN = lymph node.

The other adverse events are also summarized in Table 4. The incidences of acute GI adverse events, GU adverse events, HT and late leg edema did not differ significantly between 3DCRT and IMRT. However, the incidence of late GU adverse events higher than Grade 2 were significantly more frequently seen in the 3DCRT group (12% vs 0%, *P* = 0.038). Urinary incontinence was mostly seen as a late GU adverse event.

**Table 4.** Acute and late toxicities in 3DCRT and IMRT patients

		3DCRT (*n* = 33)		IMRT (*n* = 40)		*P*-value
		*n*	%	*n*	%	
Acute						
GI	≥G2	4	12	2	5	N.S.
	G3	0	0	0	0	N/A
GU	≥G2	3	9.1	0	0	N.S.
	G3	0	0	0	0	N/A
Hematologic	≥G2	19	58	21	53	N.S.
	G3	3	9.1	2	5	N.S.
Late						
GI	≥G2	9	27	3	7.5	0.026
	G3	6	18	0	0	0.005
GU	≥G2	4	12	0	0	0.038
	G3	0	0	0	0	N/A
Leg edema	≥G2	5	15	8	20	N.S.
	G3	2	6	1	2.5	N.S.

3DCRT = three-dimensional conformal radiation therapy, IMRT = intensity-modulated radiation therapy, GI = gastrointestinal, GU = genitourinary.

A comparison of DVH parameters for a bowel bag between 3DCRT and IMRT is summarized in Table 5. Bowel bag doses V_30_, V_40_ and V_45_ were significantly lower in IMRT than 3DCRT. However, bowel bag dose did not differ significantly between the patients who developed any grade of GI obstruction and those who did not. Additionally, the bowel bag dose did not differ significantly between the patients who developed serious GI obstruction that required surgery and those who did not.

**Table 5.** Comparisons of DVH parameters of bowel bag

	3DCRT (*n* = 33)		IMRT (*n* = 40)		*P*-value
Bowel bag	Mean	SD	Mean	SD	
V_15_ (cm^3^)	1150	356	834	292	N.S.
V_30_ (cm^3^)	1072	1120	467	164	0.049
V_40_ (cm^3^)	723	228	228	131	0.024
V_45_ (cm^3^)	680	223	192	109	0.003
Dmean (Gy)	37.4	4.72	28.9	4.05	N.S.
	Bowel obstruction (+) (*n* = 12)		Bowel obstrution (–) (*n* = 61)		*P*-value
V_15_ (cm^3^)	1187	269	936	358	N.S.
V_30_ (cm^3^)	851	222	713	867	N.S.
V_40_ (cm^3^)	660	233	441	282	N.S.
V_45_ (cm^3^)	588	278	375	290	N.S.
Dmean (Gy)	35.7	5.33	31.9	6.14	N.S.
	Bowel obstruction surgery (+) (*n* = 6)		Bowel obstruction surgery (–) (*n* = 67)		*P*-value
V_15_ (cm^3^)	1187	269	936	358	N.S.
V_30_ (cm^3^)	851	222	713	867	N.S.
V_40_ (cm^3^)	660	233	441	282	N.S.
V_45_ (cm^3^)	588	278	375	290	N.S.
Dmean (Gy)	35.7	5.33	31.9	6.14	N.S.

DVH = dose–volume histogram, 3DCRT = three-dimensional conformal radiation therapy, IMRT = intensity-modulated radiation therapy, SD = standard deviation.

## DISCUSSION

Uterine cervical cancer still is a leading cause of cancer incidence and mortality in young women worldwide. Postoperative concurrent chemoradiotherapy (CCRT) for cervical cancer decreases loco-regional recurrence and improves OS rate in patients with high risk factors after radical hysterectomy [1, 3]. However, due to concern for possible occurrence of late severe GI toxicities related to the combination of laparotomy and pelvic radiation, supported by recent emerging positive results for adjuvant chemotherapy alone for intermediate- and high-risk post-hysterectomy cervical cancer [4], several Japanese hospitals have not dared to use PORT for intermediate- and high-risk post-hysterectomy patients [5].

IMRT reduces the volume of the small bowel that receives a high dose of radiation, which potentially leads to reduction of the risks of GI obstruction, while delivering high radiation dose to the target volume. Previous reports have suggested that IMRT reduces acute or late GI adverse events [16–19]. In accordance with previous reports, the current study demonstrated that IMRT could reduce late GI adverse events, especially severe GI obstruction that requires surgical intervention. Acute GI adverse events were not significantly different between IMRT and 3DRT in this study (Table 4).

Regarding other adverse events, Chen *et al.* [17] showed that IMRT patients had a lower incidence of acute GU toxicities than 3DCRT patients. They also showed that acute HTs and late GU toxicities did not differ significantly between 3DCRT and IMRT. Isohashi *et al.* [19] showed that acute GU, late GU toxicities and late leg edema did not differ significantly between 3DCRT and IMRT groups. They showed that the IMRT patients had a significantly higher rate of acute Grade 3 hematologic toxicities than the 3DCRT patients. In the present study, the IMRT patients experienced a lower incidence of late GU toxicity greater than Grade 2, with a statistically significant difference (*P* = 0.038). Radiotherapy is known to cause a fibrotic bladder wall, resulting in a low-compliance bladder, and this is understood to be the cause of the increased incidence of urinary urgency and frequency in patients who have undergone pelvic RT [20, 21]. In this study, it was assumed that the high dose area of the bladder was reduced by IMRT, and that this led to the reduction in the incidence of late GU toxicity. There is a possibility that more patients in the IMRT group received bladder nerve sparing surgery, but this information was not taken into account in this study because the extent of bladder nerve preservation was not always written in the surgical operation records. The incidences of acute HT and late leg edema did not differ between the two radiation methods.

The radiation dose to the bowel can be a predictive factor of GI adverse events in previous reports [22–24]. In this study, we evaluated the radiation dose to the small bowel using a bowel bag as a surrogate structure. Although IMRT significantly reduced the dose to the bowel bag compared with 3DCRT, the relationship between the dose to the bowel bag and the incidence of any grade of GI obstruction or severe GI obstruction that required surgery was not clearly demonstrated in this study. It was supposed that the number of GI obstruction event was not statistically high enough for detecting a relationship between the dose to the bowel bag and the incidence of GI obstruction.

The GI obstruction rate in this study was 16.4% (12/73), and that of those who received extended-field EBRT was 28.5% (2/7). Although (possibly because of the limited number of patients with extended field) there was no statistically significant difference (*P* = 0.323), extended field might have contributed to a higher GI obstruction occurrence.

There is a concern that IMRT may increase loco-regional recurrences by reducing the target volume, in the attempt to protect the bowel. However, Previous studies have reported that the oncologic outcome did not deteriorate when using IMRT compared with conventional 3DCRT [17–19]. In accordance with these previous reports, the OS and PFS did not differ between 3DCRT and IMRT in this study. No pelvic LN recurrence was seen in either group. All the locoregional recurrences were seen in the vaginal cuff or paracolpium. Furthermore, the recurrences never occurred in the marginal zone of the RT field but in the center of the RT field in both radiation methods. The patients who had a pathologically proven vaginal invasion tend to have local recurrences more frequently. Therefore, dose escalation for the vaginal cuff or paracolpium, especially for patients who have vaginal invasion may contribute to reducing loco-regional recurrences. We consider that boost irradiation using brachytherapy or dose escalation with IMRT (simultaneous integrated boost) for the local region can be a good option for such patients.

Although since 2000 CCRT has been the standard treatment for postoperative uterine cervical cancer patients with high-risk prognostic factors [1], concomitant chemotherapy was not administered in our institution during this study period, because, as shown above, the incidence of late GI toxicity was as high as 27% with 3DCRT in our institution, which prevented us using concurrent administration of weekly cisplatin (CDDP). In this context, it can be said that our institution was in a unique position. In such special circumstances, difference between the two different radiation delivery methods alone could be investigated. To the best of our knowledge, this is the first study that has compared 3DCRT with IMRT in the setting of radiotherapy alone. Now that we have shown that IMRT was able to reduce late adverse GI toxicities, CCRT is currently performed in our institution, currently with IMRT. We will compare the outcome for RT alone and CCRT using IMRT in a future study.

There were several limitations to our study. This study was a retrospective study from a single institution with a limited number of patients. The follow-up period differed between the 3DCRT group and the IMRT group. Adjuvant RT alone is not the standard treatment for cervical cancer patients with high-risk prognostic factors after radical hysterectomy, but CCRT is the standard treatment; in this study, all patients were treated with RT alone. However, there are no reports for adjuvant RT alone that compare the efficacy and toxicity of 3DCRT and IMRT. Therefore, we consider that, although the results should be interpreted with caution, this study is important in progressing our understanding of postoperative treatment for cervical cancer.

In conclusion, our results suggested that postoperative pelvic IMRT could reduce the incidence of late severe GI obstruction and demonstrated non-inferior clinical efficacy compared with conventional 3DCRT in patients with uterine cervical cancer.

## Supplementary Material

rrz041_Supplementary_Table_1
